# Ambient Air Quality Classification by Grey Wolf Optimizer Based Support Vector Machine

**DOI:** 10.1155/2017/3131083

**Published:** 2017-08-15

**Authors:** Akash Saxena, Shalini Shekhawat

**Affiliations:** ^1^Department of Electrical Engineering, Swami Keshvanand Institute of Technology, Jaipur, India; ^2^Department of Mathematics, Swami Keshvanand Institute of Technology, Jaipur, India

## Abstract

With the development of society along with an escalating population, the concerns regarding public health have cropped up. The quality of air becomes primary concern regarding constant increase in the number of vehicles and industrial development. With this concern, several indices have been proposed to indicate the pollutant concentrations. In this paper, we present a mathematical framework to formulate a Cumulative Index (CI) on the basis of an individual concentration of four major pollutants (SO_2_, NO_2_, PM_2.5_, and PM_10_). Further, a supervised learning algorithm based classifier is proposed. This classifier employs support vector machine (SVM) to classify air quality into two types, that is, good or harmful. The potential inputs for this classifier are the calculated values of CIs. The efficacy of the classifier is tested on the real data of three locations: Kolkata, Delhi, and Bhopal. It is observed that the classifier performs well to classify the quality of air.

## 1. Introduction

Air pollution is a critical issue that influences the health of urban population. The problem becomes prominent with an exponential increase in the population and continuous industrial development. Many problems, namely, deforestation, waste management, solid waste disposal, and the release of toxic materials, contributed to and influenced the quality of air around us. With this concern, air quality assessment has become a potential area of research. The Air Quality Index is a numerical indicator used by agencies to assess the concentration of various pollutants in the air [1].  Table 1 shows the effect of various pollutants on human health.

In recent years, various methods have been developed by the researchers to assess air quality. A few of them are based on the calculation of indices. A rich survey of these indices was presented in [1]. The main objective of the index formulation is to transform the concentration of the major air pollutants into single numerical value which can be used as a representation of the air quality. Along with this, index should be easily understandable, based on the National Ambient Air Quality Standards (NAAQS).

The principal component based regression technique was proposed for the prediction of air quality in [2]. The authors developed a combination of the principal component based Multiple Linear Regression (MLR) model to forecast the air quality in Delhi [2]. A Monte Carlo dispersion model was proposed in [3]. The work addressed low wind speed meandering conditions, where the Gaussian prediction models loose relevance. The Artificial Neural Network (ANN) based prediction was performed by Elangasinghe et al. [4]. They employed ANN to predict NO_2_ concentration near Auckland Highway. The metrological variables, namely, wind speed, wind direction, solar radiation, temperature, and humidity as well as time frames, were also considered by the author to generate accurate predictions. Forecasting and prediction of troposphere ozone episodes were recently reported through MLR for Delhi [5]. A comparative study of different topologies of neural networks, namely, Layer Recurrent Neural Network (LRNN), Feed Forward Backpropagation (FFBP), and many more, was performed in the prediction of ground level ozone concentration in many researches. Different meteorological factors, namely, relative humidity, temperature, wind speed, and wind direction, were employed to forecast the ozone concentrations. It is observed that supervised learning based approaches are more relevant and accurate for forecasting the pollutant concentrations because of their ability to do parallel data processing with high accuracy and fast response and the capability to model dynamic, nonlinear, and noisy data.

It is interesting to observe that the approaches employed in this direction are based on the accuracy of the forecasting engine performance. It is also worth mentioning here that the accuracy of the forecasting engine reduces when the span of forecast increases. Environmental problems and their solutions should be based on long term forecasting of the air quality. However, there is less work reported on the classification of air quality. The classification rules are based on the numerical values of the concentration of pollutants. In [2] the concentrations of SO_2_, NO_2_, PM_2.5_, and PM_10_ were employed to derive a classification rule which can classify the quality of air and further forecast the quality. It is easy to classify the air quality on the basis of individual pollutants. However, there is an acute need of an index, which can cumulatively denote the quality of the air. In this paper numerical values of Air Quality Index (AQI) of SO_2_, NO_2_, PM_2.5_, and PM_10_ are computed and a mathematical framework is presented to form a Cumulative Index (CI). The linear discriminant functions for these mathematical combinations are decided through SVM.

On the basis of critical literature review, the following are the research objectives for this study:

1. To present a mathematical framework in the formulation of CI by employing the numerical values of Air Quality Indices of (SO_2_, NO_2_, PM_2.5_, and PM_10_)
2. To present a comparative analysis of the proposed CI with existing air quality indices
3. To establish an optimization routine and propose a solution based on Grey Wolf Optimization (GWO) algorithm by estimating the kernel parameters and bias for SVM
4. To derive a linear discriminant function for SVM to classify the data on the basis of quality of air.

In the next section, the details of CI are incorporated. This section also discusses AQI.  Section 3 describes the design of support vector machine along with the technical details of GWO.  Section 4 exhibits the numerical results along with the classification efficiency of different classifiers.  Section 5 presents the major highlights of this work in a conclusive form.

## 2. CI and Mathematical Framework

In the past, several indices have been proposed to indicate the quality of air through numerical values. Pollution index has been proposed by Cannistraro and Ponterio [10]. The index is based on the concentration of two critical pollutants. It computes the mean values of the concentration of these pollutants. The problem with this index is that it does not include concentration of all pollutants in computation. Air Quality Depreciation Index (AQDI) [11] has been proposed to determine the depreciation in the quality of air as compared to the standard values. The value of this index is not easy to calculate due to its dependence on weights. An Integral Air Pollutant Index (IAPI) has been proposed by Bezuglaya et al. [8]. The calculation of IAPI is based on subindex values of pollutants; the function used in this index is ambiguous in nature and can lead to an index in hazardous category which may be a false alarm.  Table 2 shows the calculation of this index. It has been observed that, due to its dependence on the subindex values of pollutants, the index is less sensitive to pollutant concentrations. Aggregate Air Quality Index (AAQI) has been proposed by Kyrkilis et al. [9]. Although this index is considered the effect of five pollutants at a time, the heavy computation burden on processing engines questioned the feasibility of this index. Fuzzy Air Quality Index (FAQI) has been proposed by Mandal et al. [12]. This index was based on the relationship between AQI values and output parameters but the index inherited the pitfall associated with fuzzy logic.

In this table, we have shown a few samples of pollutants concentrations from different cities of India [13].  Table 2 presents a comparative analysis of different pollution indices; it has been observed that the values of IAPI are the same for a few samples although the concentrations of pollutants are different from each other. This indicates that this index is less sensitive towards pollutant concentrations and, hence, is not able to define a crisp classification boundary for air quality. In the calculation of AAQI we have observed that, with the variation in values of *p* from 2 to 50, the values of this index fall in a narrow range. However, no clear methodology has been presented for the choice of *p*.

We have experimented with the fractional values of *p* and it has been observed that the values of this index are higher than the previous values where *p* was the integer. With these pitfalls in the existing air quality indexing system, a new index is required to draw a crisp classification boundary for an ambient air quality considering all pollutants concentrations equally at the same time.

We propose a new Cumulative Index, which has the following attributes:

- It is easy to understand and follow the NAAQS (as it is based on AQI of the pollutants).
- It does not suffer from eclipsing and ambiguity. This index can be used as an alert system as it is based on valid air quality data monitored from various air quality measurement stations located in densely populated cities of India.
- This index is computationally efficient and puts less burden on computational engines

In the work five classes *R*_1_, *R*_2_, *R*_3_, *R*_4_, and *R*_5_ are proposed on the basis of concentrations of pollutants [2]. AQI for any pollutant is given by the following mathematical equation:

(1) AQIpollutants=Ihi−IloBPollutanthi−BPollutantloCpollutant−BPollutantlo+Ilo,

where *C*_pollutant_ is the actual concentration of any pollutant, *B*_Pollutant_hi__ and *B*_Pollutant_lo__ are the high and low values corresponding to the break point *I*_hi_ is the subindex value corresponding to *B*_Pollutant_hi__, and *I*_lo_ is the subindex value corresponding to *B*_Pollutant_lo__ of the given pollutant. The range values with superscript “upper” denote the upper limit of the concentration of the pollutant. Similarly, the range values with superscript “lower” denote the lower limit. The classification rule is given below:

 
AQI_Pollutants_ ∈ *R*_1_⇒  the concentration is healthy.
 
AQI_Pollutants_ ∈ *R*_2_⇒  the concentration is considerable.
 
AQI_Pollutants_ ∈ *R*_3_⇒  the concentration is unhealthy.
 
AQI_Pollutants_ ∈ *R*_4_⇒  the concentration is highly unhealthy.
 
AQI_Pollutants_ ∈ *R*_5_⇒  the concentration is dangerous.

Now, we calculate CI for different cases where AQI_Pollutants_ belongs to different classes. In this paper we employ four major pollutant concentrations (SO_2_, NO_2_, PM_2.5_, and PM_10_). The value of CI should be such that it increases with the increase in individual pollutant and undergoes sharp growth when one or more pollutant concentrations lie in harmful range. We propose a mathematical function, which possesses both the qualities and can be used as an efficient indicator of air quality.

**Case 1 .**

Let AQI_SO_2__ ∈ *R*_1_, AQI_NO_2__ ∈ *R*_1_, AQI_PM_10__ ∈ *R*_2_, and AQI_PM_2.5__ ∈ *R*_3_ and then

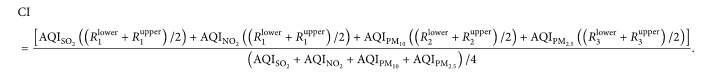
(2)

**Case 2 .**

Let AQI_SO_2__ ∈ *R*_1_, AQI_NO_2__ ∈ *R*_1_, AQI_PM_10__ ∈ *R*_3_, and AQI_PM_2.5__ ∈ *R*_2_ and then 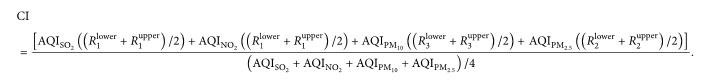
(3)

**Case 3 .**

Let AQI_SO_2__ ∈ *R*_1_, AQI_NO_2__ ∈ *R*_1_, AQI_PM_10__ ∈ *R*_3_, and AQI_PM_2.5__ ∈ *R*_4_ and then 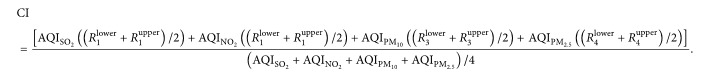
(4)

## 3. Design of Supervised Learning Model

In recent years application of SVMs in classification problems has increased due to its capability of segregation of datasets by the best hyperplane. SVMs are applied for multidimensional data classification [14], classification of microarrays [15], wind speed prediction [16], voltage stability monitoring [17], classification of power quality events [18], and contingency ranking [19]. The main reason behind this popularity of the SVMs as a classifier is that SVM can handle large feature space. The efficacy of the classification is not hindered by large dimension input feature space. However, on the other hand the efficiency of the other classifiers is dependent on the dimensions of the input feature space [17]. This distinct feature is a primary reason for the employment of the SVMs in large classification problems. Kernel functions are employed to transfer the input space of data to nonlinear high dimensional data. A sparse prediction function is generated by choosing a selected number of points and these points are support vectors (SVs). Two noteworthy features of SVMs are structural risk minimization and a fair tradeoff between empirical error and model complexity.

Let the *n* dimensional inputs *X*_*i*_  (*i* = 1,2, 3,…, *m*), where *m* is the number of samples, belong to class 1 and class 2. Associated labels are *Y*_*i*_ = 1 and *Y*_*i*_ = −1. A linear hyperplane which separates this data can be written as *f*(*x*) = 0, which can be determined by the following equation:

(5) $$\begin{array}{c} f\left(\;x\;\right)=W^{T}x+b=\overset{n}{\underset{k=1}{\sum }}W_{k}x_{k}+b, \end{array}$$

where *W* is *n* dimensional vector and *b* is scalar. These two parameters determine the location of hyperplane. The constraints are *f*(*x*_*i*_) ≥ 1 if *Y*_*i*_ ≥ 1 and *f*(*x*_*i*_) ≥ −1 if *Y*_*i*_ ≥ −1. The separating data plane which generates the maximum distance between the nearest data and the plane is called optimal separating hyperplane. Geometric margin ‖*w*‖^−2^ and insensitive loss function *ε* are the most important parameters in SVM design. Let the errors between predicted results and targets be visualized by *ε*; that is,

(6) $$\begin{array}{c} \left|\;y-f\left(\;x\;\right)\;\right|=\left\{\begin{array}{cc} 0, & \left|y-f\left(x\right)\right|\leq \varepsilon ,\\ y-f\left(x\right)-\varepsilon , & \left|y-f\left(x\right)\right|>\varepsilon . \end{array}\right. \end{array}$$

The convex optimization problem is now converted as the minimization of the geometric margin subjected to minimization of the error between predicted and simulated output; that is,

(7) J=min 12w2,subjected to y−W·fx−b−ε≤0, W·fx+b−yi≤ε, i=1,2,…,m.

Since the problems of air quality classification are data specific and depend upon the location, it is necessary to add a nonnegative slack variable *ξ*. The convex optimization problem can be converted as follows:

(8) J=min 12w2+C∑i=1mξi,subjected to y−W·fx−b≤ε+ξi, ξi≥0, i=1,2,…,m.

To find the optimal values, problem (7) can be rearranged by Lagrangian saddle point method; that is,

(9) L=12w2+C∑i=1mξi−α∑1m−y+W·fx+b+ε+ξi−∑1mηεi.

Optimization problem is solved with respect to the primal variables *W*, *b*, *ε*, and *ξ*. In general RBF is the most common choice as the mercer kernel function because of its Gaussian function form.

On the basis of ambient air quality of India reported in [13] along with the CI proposed in the previous section, we design a supervised learning based model for classification of the ambient air quality. For this, an example dataset has been created by varying the concentration of pollutants under normal distribution. The range for the concentrations is tabulated in Table 3.

**Figure 1:**
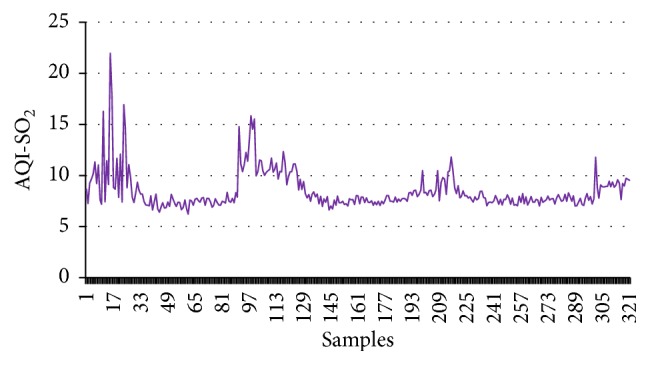
AQI-SO_2_.

**Figure 2 fig2:**
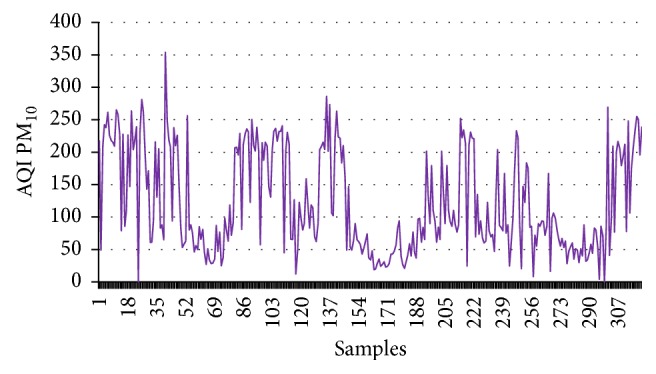
AQI PM_10_.

**Figure 3 fig3:**
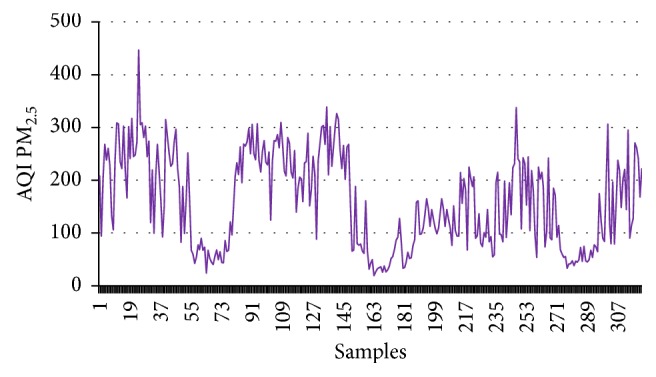
AQI PM_2.5_.

**Figure 4:**
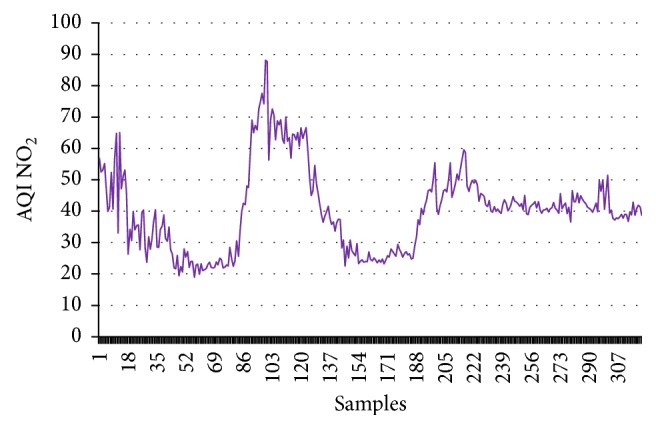
AQI NO_2_.

First this dataset has been employed to calculate CI, and then these values are employed as the input of the supervised learning model. The target data are the numerical values (1, −1) for good and harmful air quality, respectively. To train this model 1000 data points are considered; out of these data points 70% are used for training. The remaining 15-15% are used for testing and validation purpose. Figures 1 [Fig fig2] [Fig fig3]–4 show the AQI variations of SO_2_, NO_2_, PM_2.5_, and PM_10_ of example dataset. It is observed that the values taken in this set are a close replica of the concentrations as per the Indian scenario.

Table 4 shows the calculated values of CI for the example dataset. All the concentrations given in figures and tables are in *μ*g/m^3^.

It is observed that the samples exhibited in Table 4 are a close replica of actual air quality in industrial and residential areas. The following points emerged from these samples.

1. Out of 1000 samples 16 extreme cases are exhibited in this table. As per the data of air quality of Nizamuddin, Delhi, these values are realistic as the range for pollutant PM_2.5_ falls within 14–300, and for PM_10_ it is 18–890 [13]. This vast range motivates us to employ normal distribution for these two particular pollutants. The concentrations of the pollutants SO_2_ and NO_2_ fall in safer range.
2. The index proposed in this section is computationally efficient and understandable as it has high numerical value when two or more pollutants concentrations are in harmful range. It is observed from the table that the value of this index is 1159.31 when the concentrations of PM_10_ and PM_2.5_ are in harmful range.
3. It can be easily concluded that if the value of this index is higher than either single pollutant concentration is in harmful range (354.08) or two or more pollutants concentrations lie in harmful range (88.1, 215.42, and 234.23). For both of these cases values of index are 1159 and 747.
4. These observations clearly indicate that a classification boundary can be drawn with the help of numerical values of CIs. Further, with this motivation the calculations of CIs for 1000 points are conducted to build supervised learning module.

With the help of this dataset, we propose a classifier based on SVM. As it is a known fact that original SVM is a two-class separator, we employed this model to segregate air quality into two types: good and harmful. The classification rule is derived by the fact that either one of the four pollutant concentrations must lie in range *R*_3_ or higher or at least concentrations of two major pollutants lie in range *R*_2_ or higher. The kernel parameter and bias are calculated through Grey Wolf Optimizer (GWO) Algorithm. An optimization routine is established in order to calculate the kernel parameters. The aim of this optimization routine is to maximize the classification efficacy of the classifier.

(10) Maximize J, where J=Cases Classified CorrectlyTotal No. of Cases,w.r. to γ,σ,

where *γ* is regularization parameter and *σ* is kernel parameter. The following section presents the details of GWO.

## 4. Grey Wolf Optimizer

A recent population based swarm intelligence technique, called Grey Wolf Optimizer, inspired by the nature of grey wolf is discussed here. This technique was proposed by Mirjalili et al. [20] in 2014. In GWO the leadership hierarchy and the hunting behavior of grey wolf are mimicked. GWO overcomes the possibility of local optimal solutions and has greater exploration and shared information about the search space. Grey wolves are basically categorized into four groups, namely, alpha, beta, delta, and omega, for the simulation of leadership hierarchy. The three important steps of hunting, searching for prey, encircling the prey, and attacking towards prey, are employed to carry out the optimization.

Alphas are the leaders of the pack. Alphas are decision makers regarding hunting, sleeping place, time to wake up, and so forth, and that decision is followed by the pack. Hence, alpha wolf is also known as the dominant wolf. Alpha is not essentially the strongest member but good in the organization and at discipline of the pack.

Beta comes at the second level on the hierarchy of grey wolves. Betas help alpha wolves in the decision making and the activities of the pack. Betas are the best candidates to get the position of alpha in case alpha wolves pass away or become very old. The beta supports alpha's command throughout the pack.

Omega wolves have the lowest ranking in the pack. They always have to surrender to all other dominant wolves. Omega is not a main member, but, in a wolf pack, loss of an omega wolf causes the internal issues.

If a wolf does not fall in the above specified levels then he/she is delta wolf. Delta wolves have to submit before alpha and beta but they dominate omega. Scouts, elders, hunters, sentinel, and care takers belong to this group. According to Muro et al. [21] the main stages of grey wolf hunting are as follows:

- Tracking, chasing, and approaching the prey
- Pursuing, encircling, and harassing the prey
- Attacking towards the prey.

In the mathematical modeling of social hierarchy of wolf, alpha (*α*) is considered as the fittest solution, and beta (*β*) and delta (*δ*) are the second and the third best fittest solutions, respectively, in the designing of GWO. The rest of the candidates solutions are considered as omega (*ω*). The hunting is guided by *α*, *β*, and *δ*. The *ω* wolves follow *α*, *β*, and *δ* wolves. Recently the application of GWO algorithm has been conducted on Automatic Generation Control [22] and Multilayer Perceptron Training [23] and many more.

*(a) Encircling the Prey.* For the modeling of encircling the prey the following equations are proposed:

(11) D→=C→·XP→t−X→t,X→t+1=XP→t−A→·D→,

where *t* represents current iteration, $\vec{A}$ and $\vec{C}$ are coefficient vectors, $\vec{X_{P}}$ is the position vector of the prey, and $\vec{X}$ is the position vector of grey wolf.

The vectors $\vec{A}$ and $\vec{C}$ can be calculated as follows:

(12) A→=2a→·r1→−a→,C→=2·r2→.

The components of $\vec{a}$ are decreased linearly from 2 to 0 over the course of iterations and *r*_1_ and *r*_2_ are random vectors in [0,1].

*(b) Hunting for the Prey.* During hunting, the first three best solutions (*α*, *β*, and *δ*) obtained are saved and coerce the other search agents (including the omega) to update their positions according to the best search agent. The following are the proposed formulae:

(13) Dα→=C1→·Xα→−X→,Dβ→=C2→·Xβ→−X→,Dδ→=C3→·Xδ→−X→,X1→=Xα→−A1→·Dα→,X2→=Xβ→−A2→·Dβ→,X3→=Xδ→−A3→·Dδ→,X→t+1=X1+X2+X33.

**Figure 5:**
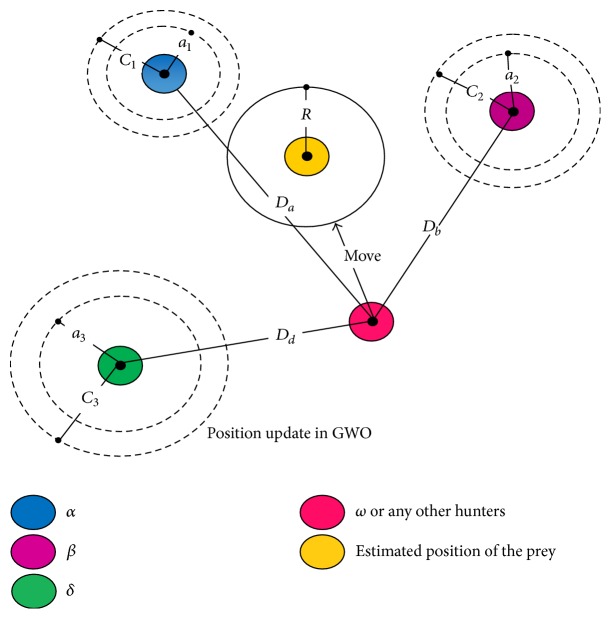
Position update in GWO [20].

Figure 5 shows the updating position of search agent according to the alpha, beta, and delta. It can be observed that alpha, beta, and delta estimate the position of the prey and other wolves update their position stochastically around the prey and the final position is random within the circle.

*(c) Attacking the Prey.* When the prey stops moving, the grey wolf finishes their hunt by attacking. Mathematically, while approaching towards the prey, the value of $\vec{a}$ decreases. Hence the fluctuation range of $\vec{A}$ is also decreased by $\vec{a}$. $\vec{A}$ is a random value in the interval [−*a*, *a*], where *a* is decreased from 2 to 0 over the course of iterations. When random values of $\vec{A}$ are in [−1,1], the next position of a search agent can be in any position between its current position and the position of the prey. If |*A*| < 1, grey wolves converge towards the prey and attack it.

*(d) Searching for the Prey.* The searching of grey wolves depends on the position of the alpha, beta, and delta. For searching, they diverge from each other. Mathematically $\vec{A}$ varies with random values greater than 1 or less than −1 to oblige the search agent to diverge from the prey. This brings out the exploration and allows GWO algorithm to search globally. If |*A* | > 1, grey wolves diverge from the prey to find the fitter prey.

### 4.1. Methodology

As described in (10) the SVM design is carried out by GWO with the aim of maximizing classification accuracy. The major parameters of this optimization process are choice of kernels, kernel parameter, and bias parameter. These parameters decide orientation and placement of the support vector in hyperplane. The GWO searches parameter space for an optimal solution by choosing proper kernels; in this work we have taken four kernels, namely, Radial Basis Function (RBF) and linear and quadratic and polynomial kernels. This optimization process has a stopping criterion of 1000 iterations along with the error tolerance 1*e* − 3. The values of parameters, calculated for population size 30, are *γ* = 10 and *σ* = 0.3 along with RBF as the choice of kernel.

**Figure 6:**
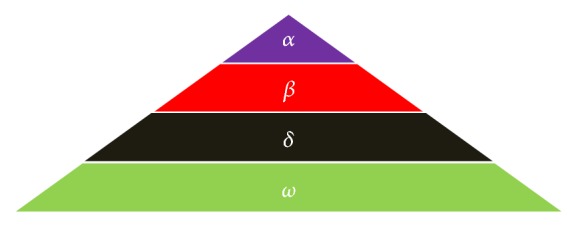
Leadership hierarchy of grey wolves [20].

Figure 6 shows leadership hierarchy of grey wolves. To see how GWO solves the optimization process few points are described below:

- The search process starts with creating a random population of grey wolves. Over the course of iterations alpha, beta, and delta wolves search for candidate solutions and in this work the solutions are in terms of choice of kernel and bias parameter and kernel parameters.
- GWO are based on the philosophy “follow the leader.” The shown hierarchy in Figure 6 assists GWO to save the best solutions obtained in terms of bias, kernel parameters, and choice of kernels over the course of iterations.
- Encircling mechanism of GWO defines a circle shaped neighborhood around the solutions, which can be extended to higher dimensions.
- Exploration and exploitation are guaranteed by adaptive values of *a* and *A*.

## 5. Simulation Results

**Figure 7:**
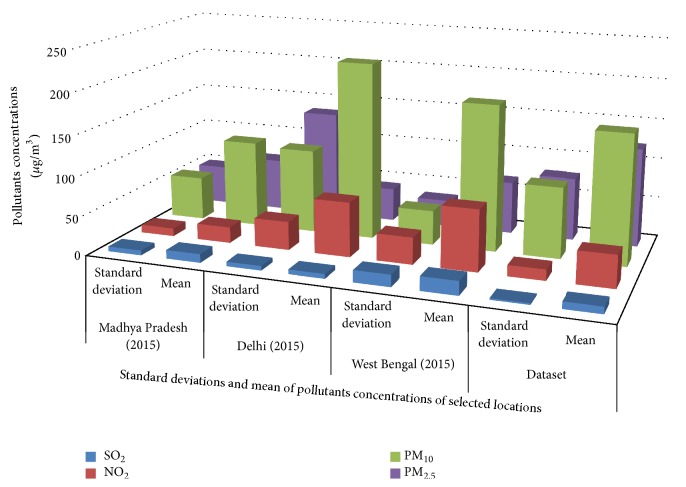
Comparative analysis of concentration of pollutants.

This section presents the classification results of air quality by the proposed supervised learning model. The efficacy of the proposed model is tested over the real data of the state of Madhya Pradesh (Bhopal), West Bengal (Kolkata), and Delhi. The historical data of ambient air quality is taken from [13]. It is observed that the proposed classifier is able to classify the quality of air. The modeling of the system and simulation studies are performed over Intel® core™, i7, 2.9 GHz 4.00 GB RAM processor unit.  Figure 7 shows the comparative analysis of different pollutant concentrations along with the example dataset.

The Central Pollution Control Board (CPCB), India, is executing a nationwide programme of ambient air quality monitoring known as National Air Quality Monitoring Programme (NAMP). The network consists of three hundred and forty-two (342) operating stations covering one hundred and twenty-seven (127) cities/towns in twenty-six (26) states and four (4) Union Territories of the country [24].

To determine the concentration of NO_2_, chemiluminescence technique is used to measure total oxides of nitrogen (NO_*x*_) by passing the sample over a heated catalyst to reduce all oxides of nitrogen to NO. Ultraviolet (UV) fluorescence technique is used to determine SO_2_ concentrations. This technique is based on the fact that SO_2_ molecules absorb UV light and become excited at one wavelength [25]. The intensity of UV light is proportional to SO_2_ concentrations. For the calculations of concentrations of SPM and RSPM Beta-Ray Absorption Light Scattering technique is used. The beta-ray absorption method is the most popular method of SPM measurement. The measurements of the beta-rays are performed with the help of beta-ray analyzer [25]. The absorption rate of beta-rays is proportional to the concentrations of SPM. An indicated value as a mass concentration is obtained from the increase of the absorption amount of beta-rays due to particles collection on filter-paper. With the enhancement of microprocessor technology, the modern gas analyzers have become sophisticated data collection nodes. These analyzers possess features like being self-automatic, self-monitoring, enabled with programmable calibration routines, and enabled with alarm circuits and are associated with high memory for data collection [25]. Tables 5, 6, and 7 show the classification results for the state of Madhya Pradesh, West Bengal, and New Delhi.

Table 5 shows the classification results of air quality of Govindpura, industrial area Akun, Bhopal (latitude is 23°.15′N and the longitude is 77°.27′E) for the year 2015. This industrial area possesses manufacturing industries, chemical industries, Grahudyog, electroplating industries, fabrication industries, and many more. Only a few samples are exhibited due to limitation of space. It is observed that, as per the results of supervised learning engine, the results with negative denominator indicate poor air quality. The values of CIs are higher: 1722 and 1568.93. It is also observed that deriving factor in this analysis is the concentration of PM_10_. Tables 5 and 6 show the results for Nizamuddin, Delhi (Residential Area) (latitude 28°35′N and longitude 77°14′E), Shahzada Bagh, Delhi (latitude is 28°.40′N and the longitude is 77°.16′E) (industrial area), and Moulali Kolkata (22°.33′N and the longitude is 88°.21′E) for year 2015.

**Figure 8:**
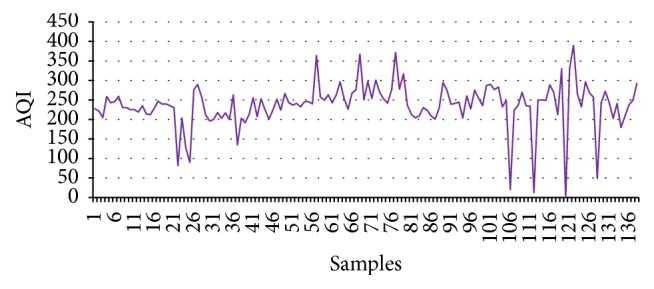
AQI of PM_10_ Delhi.

**Figure 9:**
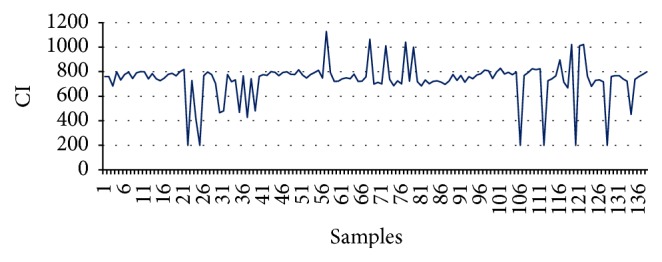
CI Delhi.

The highest concentration of PM_10_ and PM_2.5_ is the primary reason for higher values of CI in Table 6. For clarity, both the industrial area and residential areas of Delhi are chosen. Shahzadabagh industrial area houses plastic industries, clothing industries, manufacturing industries, footwear industries, and many more. Similarly, Nizamuddin area possesses vehicular pollution and it is also overcrowded. From Table 7 it can be observed that according to the values of CIs the values with negative sign are critical and concentration values of the pollutants are is also verified by the concentration values of the pollutant. It is again observed from Figures 8 and 9 that AQI of PM_10_ go along with CI.

Total 139 samples have been chosen for testing the supervised engine in case of Delhi. It can be observed from Figure 9 that pollutant concentrations are quite hazardous. It is observed that although the concentration of PM_10_ is a major driver in this calculation, the concentrations of other pollutants which are not in the safer limit contribute to the values of CIs. Hence, CI is a representative of overall concentrations of the pollutants. Moreover, the policy makers can take a collective decision on the basis of the values of CIs at different areas. For prompt actions the proposed supervised learning engine can be employed for the instant judgment of the air quality.

## 6. Conclusion

Air pollution is a major concern these days due to human health and public safety. Classification of the areas, which are harmful for respiratory system, has become a major motivation for this paper. A supervised learning model based on the example dataset has been prepared and tested over three different meteorological stations. The following are the conclusions:

- A mathematical framework for CI is proposed to employ the AQIs of different pollutants. This index is computationally efficient and understandable.
- Supervised learning model based on SVM has been developed with the help of example dataset and the values of CIs calculated. A two-class SVM has been designed. To design the SVM module GWO has been employed for parameter estimation with the aim of maximizing classification accuracy.
- The proposed architecture has been tested over the real data of Delhi, Bhopal, and Kolkata. It has been observed that the values of CIs and the classification results obtained from supervised learning model are aligned.

To develop a forecast engine for predicting the concentration on the basis of CI values lies within the scope of future work.

## Figures and Tables

**Table 1:** Major pollutants and their details.

Name of pollutant	Effect on humans	Details
SO_2_	The presence of high levels of SO_2_ has an adverse effect on human health. The exposure to high level of SO_2_ may lead to bronchitis, heart issues, respiratory illness, and asthma.	(1) This gas is an outcome of oxidation of sulphur. (2) The major source of this gas is petroleum products, coal, and volcano eruptions.

NO_2_	The presence of high levels of NO_2_ in the air leads to acid rains. This corrodes metal structures like bridges, destroys buildings, with harmful effect on aquatic life due to acid formation.	(1) This gas is an outcome of oxidation of nitrogen monoxide. (2) The major sources of NO_2_ are agricultural processes, burning of fossil fuels, biomass burning, industries, human sewage, and atmospheric deposition.

SPM (PM_2.5_) (suspended particulate matter)	The presence of high levels of SPM leads to cardiovascular diseases and respiratory issues, namely bronchitis, asthma, and lung cancer.	(1) Particulate matter is a term used for solid particles and liquid droplets found in the air. (2) Major sources of SPM are fuel combustion, power plants, and emission from diesel buses and trucks

RSPM (PM_10_) (respirable suspended particulate matter)	The presence of high levels of RSPM leads to cardiovascular diseases and respiratory issues, namely, bronchitis, asthma, and lung cancer [6, 7].	(1) The term RSPM is a composition of dust particles, industrial waste, and combustions. (2) PM10 is term used to describe tiny particles in the air, made up of a complex mixture of soot, organic, and inorganic materials having a particle size less than or equal to 10 microns' diameter.

**Table 2:** Comparative study of Air Quality Indices.

Pollutants	Concentration (*μ*g/m^3^)	AQI	IAPI [8]	AAQI [9]	CI
So_2_	2	2.5	255.28	261.81	851.85
No_2_	7	8.75			
PM_10_	264	257.21			
PM_2.5_	96	48			

So_2_	2	2.5	255.28	111.23	455.91
No_2_	11	13.75			
PM_10_	126	102.04			
PM_2.5_	84	42			

So_2_	8	10	160.94	221.33	702.69
No_2_	61	76.25			
PM_10_	158	204.5			
PM_2.5_	71	35.5			

So_2_	2	2.5	255.28	271.68	931.21
No_2_	9	11.25			
PM_10_	292	271.14			
PM_2.5_	25	12.5			

**Table 3:** Statistics of example dataset.

Pollutant (*μ*g/m^3^)	Mean	Standard deviation
SO_2_	8.49	1.90
NO_2_	40.32	13.41
PM_2.5_	159.417	87.68
PM_10_	122.12	77.64

**Table 4:** Calculation of CI.

AQI-So_2_	AQI-No_2_	AQI-PM_10_	AQI-PM_2.5_	CI
14.53	88.1	215.42	234.23	747.44
15.53	87.66	209.45	229.28	745.07
11.68	34.21	146.93	241.30	559.49
7.85	30.58	263.68	**317.37**	952.23
14.68	35.62	144	**446.54**	1127.87
8.58	31.08	61.22	219.38	475.35
8.18	36.46	93.87	99.5	199.99
6.62	31.31	**354.08**	**314.89**	1159.31
11.12	58.72	122.44	289.39	784.01
8.52	43.5	201.49	164.76	586.34
10.35	53.27	252.24	214.43	744.03
7.1	43.17	171.42	231.40	708.73
7.08	39.15	183.67	153.01	584.68
7.9	43.02	89.79	225.04	447.46
8.93	38.12	205.47	218.67	736.27
7.65	42.87	207.46	127.84	631.23

**Table 5:** Classification results of air quality data of Bhopal, Madhya Pradesh.

AQISO_2_	AQINO_2_	AQIPM_10_	AQIPM_2.5_	CI	Classification results
2.5	16.25	235.82	42	837.71	1
2.5	7.5	97	19	200	1
2.5	8.75	257.21	48	**851.84**	−1
2.5	12.5	230.84	13.5	**913.86**	−1
2.5	25	413.96	43	***1568.93***	−1
2.5	16.25	214.43	93	727.23	1
2.5	11.25	271.14	12.5	**931.21**	−1
2.5	13.75	102.04	42	455.91	1
2.5	20	252.73	36	**851.25**	−1
2.5	10	207.46	4.5	**941.26**	−1
2.5	18.75	367	31.5	**1250.94**	−1
2.5	13.75	216.42	32.5	**854.55**	−1
2.5	16.25	406.42	2.5	***1722.52***	−1
2.5	6.25	282.09	10	**952.01**	−1
2.5	22.5	202.98	8.5	**888.39**	−1
2.5	21.25	233.83	65	781.35	1
2.5	11.25	222.39	13	**915.89**	−1
2.5	11.25	209.45	9	**923.42**	−1
2.5	20	305.30	49	**1173.91**	−1

**Table 6:** Classification results of air quality data of Kolkata, West Bengal.

AQISO_2_	AQINO_2_	AQIPM_10_	AQIPM_2.5_	CI	Classification results
2.5	32.5	37	10.5	200	1
20	107	236.81	51	761.56	1
23.75	114	227.86	48.5	752.01	1
10	76.25	204.48	35.5	702.69	1
6.25	61.25	4.08	28.5	200	1
5	68.75	191.83	38.5	453.60	1
16.25	109	228.36	65.5	741.58	1
26.25	138	339.73	184.89	**981.20**	−1

**Table 7:** Classification results of air quality data of Delhi.

AQISO_2_	AQINO_2_	AQIPM_10_	AQIPM_2.5_	CI	Classification results
5	53.75	364.13	49	**1127.53**	−1
5	57.5	267.16	80.5	722.39	1
5	55	276.12	61	757.63	1
5	83.75	367	55	**1063.69**	−1
5	68.75	250.25	77.5	699.87	1
5	81.25	297.01	81.5	712.53	1
5	76.25	254.23	72	700.37	1
5	72.5	301	67.5	**1011.21**	−1
5	77.5	269.65	50.5	737.09	1
5	76.25	252.24	82.5	686.30	1
5	65	241.79	57.5	725.10	1
5	76.25	276.12	85	700.59	1
5	78.75	371.30	75.5	**1041.21**	−1
5	75	278.11	69	722.21	1
5	77.5	316.78	77	**999.46**	−1
5	61.25	236.81	63	718.83	1
5	62.5	248.75	36	766.35	1
5	75	288.55	242.01	**896.91**	−1
5	73.75	269.65	71	715.64	1
5	66.25	211.94	80.5	667.36	1
5	66.25	329.69	81	**1022.27**	−1
5	80	329.69	74	**1010.92**	−1
5	86.25	389.95	174.83	**1021.61**	−1
